# Protective effects of acerola juice on genotoxicity induced by iron *in vivo*

**DOI:** 10.1590/1678-4685-GMB-2015-0157

**Published:** 2016

**Authors:** Roberta Nunes Horta, Vivian Francilia Silva Kahl, Merielen da Silva Sarmento, Marisa Fernanda Silva Nunes, Carem Rejane Maglione Porto, Vanessa Moraes de Andrade, Alexandre de Barros Falcão Ferraz, Juliana Da Silva

**Affiliations:** 1 Centro de Ciências da Saúde, Universidade da Região da Campanha (URCAMP), Bagé, RS, Brazil; 2 Laboratório de Genética Toxicológica, Universidade Luterana do Brasil, Canoas, RS, Brazil; 3 Laboratório do Farmacognosia e Fitoquímica, Universidade Luterana do Brasil, Canoas, RS, Brazil; 4 Laboratório de Biologia Celular e Molecular, Programa de Pós-Graduação em Ciências de Saúde, Unidade de Ciências de Saúde, Universidade do Estado de Santa Catarina, Criciúma, SC, Brazil

**Keywords:** genotoxicity, comet assay, micronucleus, acerola juice, iron

## Abstract

Metal ions such as iron can induce DNA damage by inducing reactive oxygen species (ROS) and oxidative stress. Vitamin C is one of the most widely consumed antioxidants worldwide, present in many fruits and vegetables, especially in*Malpighia glabra* L., popularly known as acerola, native to Brazil. Acerola is considered a functional fruit due to its high antioxidant properties and phenolic contents, and therefore is consumed to prevent diseases or as adjuvant in treatment strategies. Here, the influence of ripe and unripe acerola juices on iron genotoxicity was analyzed *in vivo* using the comet assay and micronucleus test. The comet assay results showed that acerola juice exerted no genotoxic or antigenotoxic activity. Neither ripe nor unripe acerola juices were mutagenic to animals treated with juices, in micronucleus test. However, when compared to iron group, the pre-treatment with acerola juices exerted antimutagenic activity, decreasing significantly micronucleus mean values in bone marrow. Stage of ripeness did not influence the interaction of acerola compounds with DNA, and both ripe and unripe acerola juices exerted protective effect over DNA damage generated by iron.

## Introduction

Fruits play a prominent role in the prevention of various diseases, such as cancer, cardiovascular and neurodegenerative conditions. People who eat fruits in childhood have 38% less probability to develop cancers (Maynard *et al.*, 2003) and balanced diets can contribute for the genomic stability (Fenech, 2014). More attention has been paid to these foods, since epidemiological evidence has shown that regular consumption of vegetables is associated with reduced mortality and morbidity from some chronic diseases (Strandhagen*et al.*, 2000), and its protective effect has been attributed to the presence of constituents with antioxidant properties such as pholyphenols, carotenoids, and vitamins (Nunes*et al.*, 2011; Kahl*et al.*, 2012; Liu, 2013; Kozlowska and Szotask-Wegierek, 2014).

*Malpighia glabra* L., popularly called "acerola" in Brazil or "Barbados cherry", is a native species from tropical America. Acerola compounds, such as vitamin C, carotenoids, precursors of vitamin A, lycopene, among others (Chaves *et al.,* 2004), depends on the cultivars, environmental conditions and the stage of fruit ripeness (Chaves *et al.*, 2004;Nunes *et al.*, 2011).

Despite scientific data reporting benefits from juice consumption (Franke *et al.*, 2006; Leffa *et al.*, 2013), some compounds present in juices have also been identified as being mutagenic or carcinogenic (Spada *et al.*, 2008). Although the genotoxicity of several components of acerola juice have already been evaluated individually, it is important to test the effect of whole juice, a complex mixture, in different biological systems. Genotoxic effects, for example, may be mediated by the interaction of juice compounds with transition metals or by-products of juice auto-oxidation; Vitamin C can act as a pro-oxidant, because its reducing ability, through Fenton and Fenton-like reactions (De Freitas and Meneghini, 2001).

One feature of the normal human diet is the simultaneous presence of both essential and toxic metals (Beyersmann and Hartwig, 2008). Metal ions generate DNA damage directly or indirectly by formation of reactive oxygen species (ROS) (De Freitas and Meneghini, 2001; Phaniendra *et al.*, 2015). In recent years, many studies have been conducted on the role of ROS in the etiology of various diseases since some free radicals, in a condition of oxidative stress, are not neutralized by antioxidant cell protective mechanisms or antioxidant compounds (Halliwell and Guterridge, 2000; Phaniendra *et al.*, 2015). Vitamin C, present in acerola juice is an example of an antioxidant compound that can chelate metals, thus preventing the generation of ROS (De Freitas and Meneghini, 2001; Phaniendra *et al.*, 2015), apart from playing a role in the regulation of DNA repair enzymes (Jomova and Valko, 2011). Considering the importance of consumption of fruits and the intense growth of the culture of acerola in Brazil and its widespread use, the aim of this study was to test the genotoxic effects of acerola juice at two ripeness stages associated with metallic agents *in vivo* in mice, using the comet assay and the micronucleus test, in order to improve our understanding of the role of dietary antimutagens and anticarcinogens in humans.

## Material and Methods

### Preparation of acerola juice

Acerola samples were collected from an organic culture in May 2008, in the district of Ubajara, CE, Brazil. The fruits were collected randomly across the whole plant, and were sorted according to ripeness using a skin color gradient, as follows: (1) green fruits (unripe), fruits showing a green hue on more than 75% of its skin; and (2) red fruits (ripe), those showing a red or burgundy hue on 100% of the skin (Campelo *et al.*, 1998). After collection, fruits were kept frozen and protected from light in order to preserve their chemical and physical characteristics upon juice preparation (Nunes*et al.*, 2011). Approximately 20 units of acerola fruit were used to produce 150 g of pulp extract, after fruits were peeled and seeds removed. The pulp extract from ripe and unripe fruits were diluted with water (150 g of pulp extract per liter of water), forming the juice used in treatments. A glass of juice consumed by humans usually has 200 mL, and therefore, is composed by around 30 g of pulp extract. The acerola cultivar used in this study was AC-69.

### Animals and treatment

Animals used were CF1 mice *Mus musculus*, aged 5-7 weeks old, weighing 20-40 g, provided by the Fundação Estadual de Produção e Pesquisa em Saúde (FEEPS), Porto Alegre, RS, Brazil. The animals were kept in the central animal house of Lutheran University of Brazil (ULBRA), Canoas, RS, Brazil. The temperature in the experimental room was about 24 °C, and relative humidity was roughly 60%. The light cycle was 12 h light/12 h dark. All animals received commercial standard mouse cube diet (Labina-PURINE) and water *ad libitum*. All experimental procedures were approved by CONEP 2008-008-A, Brazil (National Commission of Ethics in Research).

Ten mice per group (five females and five males) were used for the treatment (0.1 mL/10 g body weight) divided in six groups: (a) Negative control, water; (b) Positive Control, FeSO_4_ (35 mg/kg), (c) Unripe acerola juice; (d) Ripe acerola juice; (e) Unripe acerola juice plus FeSO_4_; (f) Ripe acerola juice plus FeSO_4._ The dose, exposure time and the administration route of FeSO_4_ were based on a previous study (Nunes *et al.*, 2011). Table 1 summarizes the treatment period. Acerola, FeSO_4_ and water were administrated by gavage. The test substance was handled as potentially mutagenic, according to the safety procedures required.

**Table 1:** Experimental procedures: treatment protocols and blood sampling schedules.

Group	Exposure and sampling schedule				
	0h	3h	24h		48h
Control	Treatment: water	Blood sampling	Blood sampling	Treatment: water	Blood and bone marrow sampling
FeSO_4_	Treatment: FeSO_4_	Blood sampling	Blood sampling	–	Blood and bone marrow sampling
Unripe acerola juice	Treatment: unripe acerola juice	Blood sampling	Blood sampling	Treatment: unripe acerola juice	Blood and bone marrow sampling
Ripe acerola juice	Treatment: ripe acerola juice	Blood sampling	Blood sampling	Treatment: ripe acerola juice	Blood and bone marrow sampling
Unripe acerola juice + FeSO_4_	Pre-treatment: unripe acerola juice	–	–	Treatment: FeSO_4_	Blood and bone marrow sampling
Ripe acerola juice + FeSO_4_	Pre-treatment: ripe acerola juice	–	–	Treatment: FeSO_4_	Blood and bone marrow sampling

### Genotoxicity assays

#### Comet assay

The analysis was conducted in accordance with the protocol described by Singh *et al.* (1998)with some modifications (Nunes *et al.,* 2011). Blood cells (10 μL of whole blood with heparin) were embedded in 90 μL of 0.75% (w/v) low melting point agarose and the mixture added to a microscope slide pre-coated with 1.5% (w/v) of normal melting point agarose and topped with a coverslip. The slides were briefly placed on ice for agarose to solidify and then the coverslips were carefully removed. Next, the slides were immersed in lysis solution (2.5 M NaCl, 100 mM EDTA and 10 mM Tris, pH 10.0-10.5) containing freshly added 1% Triton X-100 and 10% dimethyl sulfoxide (DMSO) for at least 1 h at 4 °C. Subsequently, the slides were incubated in freshly made alkaline buffer (300 mM NaOH and 1 mM EDTA, pH *>* 13) for 20 min for DNA unwinding, and electrophoresis was performed in the same buffer. The electrophoresis conditions were 15 min at 300 mA and 25 V (0.7 V/cm). All these steps were carried out under dim indirect light. Following electrophoresis, slides were neutralized in 400 mM Tris buffer (pH 7.5) and fixed (15% w/v trichloroacetic acid, 5% w/v zinc sulfate, 5% glycerol), washed in distilled water and dried overnight. The gels were re-hydrated for 5 min in distilled water, and then stained for 15 min (37 °C) with a solution containing the following sequence: 34 mL of Solution B (0.2% w/v ammonium nitrate, 0.2% w/v silver nitrate, 0.5% w/v tungstosilicic acid, 0.15% v/v formaldehyde, 5% w/v sodium carbonate) and 66 mL of Solution A (5% sodium carbonate). The staining was stopped with 1% acetic acid and the gels were air-dried. To calculate a damage index (DI), cells were visually allocated into 5 classes according to tail size (0 *=* no tails, to 4 *=* maximum-length tails) which resulted in a single DNA damage score for each sample and consequently for each group studied. Thus, the damage index (DI) of the group could range from 0 (completely undamaged = 100 cells X 0) to 400 (maximum damage =100 cells X 4). The damage frequency (DF in %) was calculated for each sample based on the number of cells with tail *vs.* those without. All slides were coded for blinded analysis.

The results of the potential of acerola juice to modulate the effect of FeSO_4_ treatment were expressed as described in the literature (Kapiszewska *et al.,*2005), as percentage inhibition of damage index (DI) according to the expression: (I%): percentage of inhibition of DI = [FeSO4 DI - DI of the extract with FeSO_4_] / [FeSO_4_ DI - DI negative control] x 100

#### Micronucleus test

Each complete test was made according to a report by Mavournin *et al.* (1990) and OECD (2013). Bone marrow smears were prepared for the 48-h exposure sample, when animals were killed by decapitation. The bone marrow was extracted from the two femurs and the smears were prepared directly on slides, two per animal, with a drop of fetal calf serum. The slides were stained with 10% Giemsa for 5 min, air-dried and coded for blinded analysis. To avoid false negative results and as a measure of toxicity in bone marrow, the polychromatic erythrocytes: normochromatic erythrocytes (PCE/NCE) ratio was scored in 1,000 cells. The incidence of micronuclei was observed in 2,000 PCE for each animal (*i.e.* 1,000 from each of the two slides prepared from the duplicate), using bright-field optical microscopy at a magnification of 200–1000. The test groups were compared to the respective negative controls by gender, separately and in combination.

### Statistical analysis

The normality of variables was evaluated using the Kolmogorov–Smirnov test. Statistical differences between the groups were analyzed using the non-parametric two-tailed Kruskal–Wallis Test with the Dunn correction for multiple comparisons for comet assay and micronucleus test results. Student's*t*-test was used to compare damage between genders. The critical level for rejection of the null hypothesis was considered to be a*P* value of 5%.

## Results

Table 2 summarizes the comet assay data expressed as damage index (DI) and damage frequency (DF) for blood cells of mice exposed for 3 h, 24 h and 48 h to water, FeSO_4_ and acerola juices, at different maturation stages. The FeSO_4_ group showed a significant increase in DI, as compared to the negative control, at 24 h (P < 0.01) and 48 h (P < 0.001). After treatment with FeSO_4_ at 48 h, the mean DF values were also significantly elevated (P < 0.001). Concerning the acerola juice groups, no genotoxic activity was observed, when compared with the negative control group.

**Table 2:** Comet assay parameters (damage index and damage frequency index; mean ± standard deviation) for blood samples of mice treated with unripe and ripe acerola juice. For each group, n = 10 (five males and five females), with 100 cells per animal.

Groups	Gender	Schedule^a^					
		3h		24h		48h	
		Per Gender	Per Group	Per Gender	Per Group	Per Gender	Per Group
Damage Index Water	Male	7.60 ± 3.43	6.70 ± 3.94	7.80 ± 1.30	8.60 ± 2.79	10.80 ±3.27	8.50 ± 4.03
	Female	5.80 ± 4.60		9.40 ± 3.78		6.20 ± 3.56	
FeSO_4_	Male	10.00 ± 4.89	10.60 ± 6.39	19.50 ± 1.92	32.13 ±28.80^b^	41.00 ±4.96	36.00 ± 10.70^c^
	Female	11.20 ± 8.20		44.75 ± 38.86		31.00 ± 13.29	
Unripe acerola juice	Male	3.80 ± 3.03	4.60 ± 3.37	14.00 ± 8.66	13.33 ±7.84	2.60 ± 3.71	2.70 ± 3.83
	Female	5.40 ± 3.84		12.50 ± 7.59		2.80 ± 4.38	
Ripe acerola juice	Male	6.80 ± 6.05	5.10 ± 4.65	14.00 ± 5.78	13.90 ±3.98	2.80 ± 2.28	2.40 ± 1.89
	Female	3.40 ± 2.19		13.80 ± 1.48		2.00 1.58	
Damage Frequency Water	Male	6.40 ± 2.60	5.00 ± 3.27	4.80 ± 2.49	5.00 ± 2.35	5.40 ± 1.81	4.30 ± 2.05
	Female	4.80 ± 3.96		5.20 ± 2.49		3.20 ± 1.78	
FeSO_4_	Male	7.80 ± 3.63	6.67 ± 3.50	9.20 ± 1.00	17.13 ± 18.13	28.00 ±4.24	25.38 ± 8.71^c^
	Female	5.25 ± 3.20		24.75 ± 24.72		22.75 ± 11.87	
Unripe acerola juice	Male	2.60 ± 1.94	3.10 ± 1.96	11.60 ± 4.98	10.68 ±4.89	1.60 ± 2.30	1.50 ± 2.12
	Female	3.60 ± 2.07		9.75 ± 5.13		1.40 ± 2.91	
Ripe acerola juice	Male	4.80 ± 4.43	3.00 ± 3.55	9.20 ± 3.27	10.00 ±3.39	1.60 ± 1.14	1.60 ± 1.17
	Female	1.20 ± 0.83		10.80 ± 3.70		1.60 ± 1.34	

a For more details see Table 1.

b Significant difference in relation to water (negative control) at P < 0.01, Kruskall-Wallis test.

c Significant difference in relation to water (negative control) at P < 0.001, Kruskall-Wallis test.

**Figure 1:**
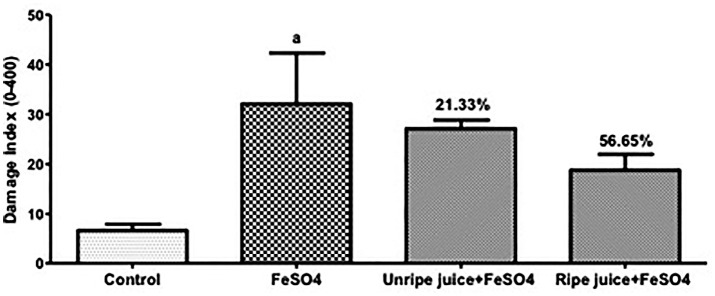
Modulation of DNA damage induced by FeSO_4_ by pre-treatment with unripe (21.33%) and ripe acerola (56.65%) juices evaluated by Comet assay. ^a^Significant compared to control group at P < 0.001.

Figure 1 shows that groups pre-treated with acerola presented lower DI than the FeSO_4_ group. Nevertheless, no statistically significant difference in antigenotoxic activity of acerola juice was observed in relation to FeSO_4._ The pre-treatment with unripe acerola juice presented modulation of DNA damage of 21.33%, while the modulation by ripe acerola juice was 56.65%. Our study also found no differences between juice maturation stage and animal gender in the comet assay results.

Table 3 shows the results of the micronucleus test for bone marrow samples. Bone marrow cells of mice treated with unripe and ripe acerola juice showed no significant increase in micronucleus mean, when compared with negative control, therefore not presenting mutagenicity. FeSO_4_ was mutagenic when compared to water (P < 0.001) and acerola juices from ripe (P < 0.01) and unripe (P < 0.001) fruits. There was no significant difference in PCE/NCE ratios, indicating the absence of toxicity. No difference in micronuclei formation was observed between genders.

**Table 3:** Detection of micronucleus mean (± standard deviation) in bone marrow polychromatic erythrocytes (MNPCE) of mice individuals exposed to acerola juice. Each group, n = 10 (five males and five females), with 2000 cells/animal.

Groups	Gender	Bone marrow (MNPCE)		Ratio (PCE:NCE)	
		Per gender	Per group	Per gender	Per group
Water	Male	1.80 ±0.83	2.50 ± 1.35	1.09 ± 0.28	1.14 ± 0.29
	Female	3.20 ± 1.48		1.19 ± 0.32	
FeSO_4_	Male	7.33 ± 1.52	7.28 ± 1.49^a^, ^b^, ^c^	1.18 ± 0.27	1.26 ± 0.23
	Female	7.25 ± 1.70		1.33 ± 0.21	
Unripe acerola juice	Male	2.00 ± 1.00	1.90 ± 0.87	0.86 ±0.11	0.91 ± 0.13
	Female	1.80 ± 0.83	0.95 ±0.14		
Ripe acerola juice	Male	4.60 ± 1.14	4.00 ± 1.70	0.94 ± 0.23	0.93 ± 0.16
	Female	3.40 ±2.07		0.92 ± 0.09	

a P < 0.001 in relation to unripe acerola juice, Kruskall-Wallis test;

b P < 0.01 in relation to ripe acerola juice, Kruskall-Wallis test.

c Significant at P < 0.001 in relation to water, Kruskall-Wallis test.

**Figure 2:**
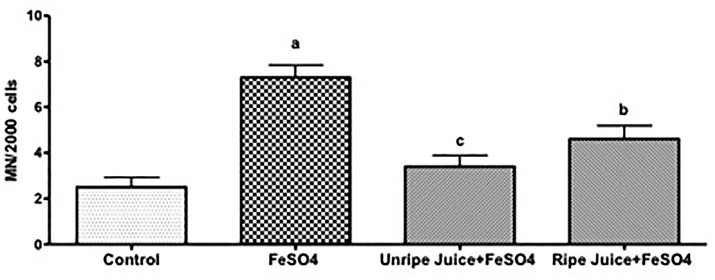
Antimutagenic activity of acerola unripe and ripe juices evaluated by Micronucleus test. ^a^Significant compared to control group at P < 0.001; ^b^significant compared to FeSO_4_ group at P < 0.01, and ^c^P < 0.001; Kruskall-Wallis (Dunn).

Bone marrow cells of mice pre-treated with juices from unripe and ripe acerola fruits showed a significant decrease in micronucleus (P < 0.001 and P < 0.01, respectively), when compared with the FeSO_4_ group, revealing antimutagenic activity (Figure 2).

## Discussion

A recent study considers acerola as a functional food since its pulp shows great antioxidant activity and phenolic content and, therefore is consumed to prevent diseases or as adjuvant in treatment strategies (Paz*et al.*, 2015). Acerola shows complex traits, interacting with biological organisms in many ways. In this study, genotoxicity and antigenotoxicity of acerola juices in different stages of ripeness in relation to FeSO_4_ DNA damages were evaluated in blood cells of mice using the comet assay and in bone marrow cells using the micronucleus test. The choice of these two types of fruit stage is due to the fact that the physicochemical characteristics of acerola undergo changes as the fruit ripens (Chaves *et al.*, 2004; Nunes *et al.*, 2011; Paz *et al.*, 2015).

Acerola juice from unripe or ripe juices did not show genotoxic and mutagenic potential in mice after *in vivo* treatment. Our previous studies have shown that acerola samples of lyophilized fruit pulp collected in two Brazilian states (São Paulo and Ceará) were not genotoxic in mice blood cells *ex vivo* (Nunes *et al.*, 2012). A similar result was observed for genotoxicity *ex vivo* also when two ripeness stages of acerola were used (Nunes *et al.*, 2011). The genotoxicity of juices of different fruits and vegetables, such as orange, guava,*Pterodon emarginatus* and carrot has been studied, also showing no genotoxicity (Franke *et al.*, 2006; Fernandéz-Bedmar *et al.*, 2011; Lee *et al.*, 2011).

Iron plays an essential role in metabolism, since it participates in the transport of oxygen and xenobiotic metabolizing enzymes (De Freitas and Meneghini, 2001; Cooper and Groves, 2011). However, exposure to excessive amounts of iron can damage cells and organs, since the metal takes part in reactions which generate reactive oxygen species (ROS) (Franke *et al.*, 2006; Beyersmann and Hartwig, 2008; Jomova and Valko, 2011). The World Health Organization (WHO, 2002) has established as the recommended dietary allowance (RDA) between 8 and 18 mg/day iron, depending on the gender. The tested dose in this study corresponds to about 12% of the LD_50_ for FeSO_4_ and was genotoxic 24 h after treatment, inducing DNA damage in the comet assay (24 h and 48 h) and micro-nucleus test (48 h). The evolution of systemic effects and the peak of body iron intoxication occur 4-6 h after administration of large quantities of the compound (SACN, 2010), which can justify the non-genotoxic effect 3-h after exposure. It is likely that the DNA damage was generated early after exposure, as a consequence of the iron peak in the blood and inside the cells. Despite the DNA damage observed after 24 h and 48 h, it is not clear whether iron was free or bound in blood. Although free iron is not likely to exist in biological systems, it is well known that the presence of trace amounts of "free" iron (*i.e.*, weakly bound) is involved in the generation of oxidative stress (Koskenkorva-Frank *et al.*, 2013). Similarly to the present work, a previous study demonstrated analogous results in mice exposed to FeSO4 in the comet assay (Franke *et al.,* 2006).

When pre-treatment was evaluated, acerola juice in both maturation stages did not significantly reduce DNA damage evaluated by the comet assay, though unripe and ripe acerola juices showed an interesting modulation of 21.33% and 56.65%, respectively, in relation to FeSO4 treatment only, apart from antimutagenic activity in the micronucleus test. Thus, pre-treatment with acerola juice protected the blood cells against DNA damage generated by FeSO4 Although the main positive aspect of acerola fruit is its high vitamin C content, it also presents large quantities of antioxidants, such as carotenoids, flavonoids, and anthocyanins (Chaves *et al.,* 2004; Nóbrega *et al.,* 2014; Silva *et al.,* 2014). Some studies stated that the antioxidant potential of acerola juice depended on its content of phenolic compounds and vitamin C (Leffa*et al.,* 2013; Dias*et al.,* 2014; Paz*et al.,* 2015). Vitamin C has been studied for its protective action against different diseases (Folchetti *etal.*, 2014; Ives *et al.,* 2014; Koike *et al.,* 2014). The mechanisms by which vitamin C acts include antioxidant activity as well as bio-antimutagenic and/or desmutagenic actions (De Freitas and Meneghini, 2001; Franke *et al.,* 2006). Although many other factors are involved in the differences observed, considerable evidence demonstrated that acerola juice has beneficial potential to DNA, protecting it against damages caused by FeSO_4_.

In a previous study from our laboratory (Nunes*et al.,* 2011) we observed that ripe acerola showed antigenotoxicity activity against damage caused by hydrogen peroxide *ex vivo.* In the present study, we observed modulation of damage caused by FeSO_4_ to both unripe and ripe acerola juice. Furthermore, pre-treatment showed significant antimutagenic activity. Despite the differences in antigenotoxic activities between unripe and ripe fruits (Nunes *et al.,* 2011), in the present study we did not observe this effect, probably due to the biological response of metabolism*in vivo* and the complex mixture of nutrients of acerola juice. The chemical composition of fruits evaluated by HPLC (the same samples as in the previous study) demonstrated that unripe acerola showed higher levels of vitamin C (8,104 mg/100 g of acerola sample) than ripe acerola (4,447.6 mg/100 g of acerola sample). Quantification analysis shows that the ripe acerola has a higher content of flavonoids (15.3 ± 0.35%) in comparison to unripe acerola (8.7 ± 0.21%). For rutin and quercetin no significant differences were observed (150 mg of rutin/100 g of acerola sample and 57 mg of quercetin/100 g of acerola sample). It was also observed in this previous study (Nunes *et al.,* 2011) that the amount of extract needed to capture the DPPH free radical is lower in unripe than in ripe acerola, and that the antioxidant capacity of unripe fruit at IC50 was two times higher than that of ripe sample. In conclusion, both unripe and ripe acerola juice exerted protective effect on DNA damage generated by FeSO_4_. Stage of ripeness did not influence the interaction of acerola compounds with DNA, showing that consumption of acerola juice, combined with a healthy and balanced diet, can lead to a protective effect.
